# Marine Bioactive Peptides for Colorectal Cancer Therapy: Mechanisms, Therapeutic Potential, and Translational Challenges

**DOI:** 10.3390/md24050170

**Published:** 2026-05-09

**Authors:** Yueyang Lu, Guixiao Wang, Mei Zhou, Tianbao Chen, Zhimin Fan

**Affiliations:** 1 Jiangsu Clinical Innovation Center for Anorectal Diseases of T.C.M., Nanjing Hospital of Chinese Medicine Affiliated to Nanjing University of Chinese Medicine, Nanjing 210022, China; ylu21@qub.ac.uk; 2 School of Pharmacy, Queen’s University Belfast, 97 Lisburn Road, Belfast BT9 7BL, UK; gwang13@qub.ac.uk (G.W.); m.zhou@qub.ac.uk (M.Z.); t.chen@qub.ac.uk (T.C.)

**Keywords:** marine bioactive peptides, colorectal cancer, anti-colorectal cancer mechanism, translation

## Abstract

Colorectal cancer (CRC) is one of the most prevalent gastrointestinal malignancies worldwide and remains a major cause of cancer-related mortality. Although current treatment strategies, including surgery, chemotherapy, radiotherapy, and targeted therapies, have improved patient outcomes, their effectiveness is frequently limited by multidrug resistance, severe adverse effects, tumour recurrence, and restricted patient applicability. Consequently, there is an urgent need to develop novel therapeutic agents with improved efficacy and reduced toxicity. Marine bioactive peptides have emerged as promising candidates for CRC therapy because of their remarkable structural diversity, unique evolutionary adaptations, and broad spectrum of biological activities. Numerous marine-derived peptides exhibit potent anti-CRC effects by inducing apoptosis, regulating cell-cycle progression, suppressing invasion and metastasis, inhibiting angiogenesis, and modulating the tumour microenvironment while generally demonstrating low toxicity toward normal cells. Despite these advantages, the clinical translation of marine peptides remains constrained by several challenges, including poor stability, rapid enzymatic degradation, limited bioavailability, difficulties in large-scale production, insufficient target characterization, and a lack of long-term safety evaluation. Recent advances in peptide engineering and pharmaceutical technology have significantly accelerated progress in this field. Strategies such as structural modification, cyclization, nanoformulation, intelligent delivery systems, and artificial intelligence-assisted peptide design have improved peptide stability, targeting efficiency, pharmacokinetic properties, and production feasibility. These technological innovations provide new opportunities to overcome the major limitations associated with marine peptide therapeutics. This review systematically summarizes the sources, structural characteristics, extraction and purification methods, molecular mechanisms, and in vitro and in vivo anti-CRC activities of marine-derived peptides. In addition, the major translational challenges and current technological solutions are critically discussed, with particular emphasis on the integration of multidisciplinary approaches for the development of next-generation marine peptide-based therapeutics for colorectal cancer.

## 1. Introduction

Colorectal cancer (CRC) is a gastrointestinal malignancy with persistently high global incidence and mortality, posing a major public health challenge. Epidemiological data indicate that in 2022, there were over 1.9 million new cases and 900,000 deaths worldwide, with CRC ranking as the third most commonly diagnosed cancer and the second leading cause of cancer death. Driven by population growth and ageing, the disease burden is projected to worsen, with an anticipated 3 million new cases and 1.6 million deaths by 2040 [1,2,3].

Current primary treatments for CRC include surgery, chemotherapy, radiotherapy, immunotherapy, and targeted therapy. Surgery is the preferred option for patients with resectable early-stage disease, but it only addresses local lesions and is not curative. It also carries risks such as blood loss, infection, and thrombosis [4]. Patients with unresectable tumours rely on combined regimens like chemotherapy and radiotherapy, which have significant drawbacks. Common chemotherapy agents like 5-fluorouracil and oxaliplatin often damage the digestive and hematopoietic systems. Furthermore, cancer cells develop multidrug resistance, frequently through the overexpression of P-glycoprotein, leading to acquired resistance in over half of patients on combination therapy [5]. Radiotherapy causes rectal discomfort and abnormal defecation, while both surgery and radiotherapy may, paradoxically, increase the risk of metastasis. In the realm of immunotherapy for CRC, PD-1/PD-L1 immune checkpoint inhibitors (ICIs), such as pembrolizumab and nivolumab, have been approved for patients with mismatch repair deficiency (dMMR) or high microsatellite instability (MSI-H). However, only around 5% of metastatic CRC cases belong to this specific molecular subtype. This implies that the majority of patients with proficient mismatch repair (pMMR) or microsatellite stable (MSS) tumours exhibit insensitivity to ICI therapy. Additionally, ICI administration often results in immune-related adverse events (irAEs), including diarrhea, rash, and colitis. In the domain of targeted therapy, EGFR-directed agents such as cetuximab and panitumumab are strictly indicated for RAS/BRAF wild-type metastatic CRC patients. Bevacizumab exerts its antitumour effect by inhibiting vascular endothelial growth factor (VEGF)-mediated angiogenesis, with its application independent of RAS/BRAF mutation status. For the approximately 10% of metastatic CRC patients harbouring the BRAF V600E mutation, BRAF inhibitors like encorafenib provide a targeted therapeutic option. These patients typically demonstrate poor response to conventional chemotherapy and unfavourable prognosis, whereas encorafenib improves their clinical outcomes. Nevertheless, patients frequently develop acquired resistance within months of initiating targeted therapy. This resistance is commonly driven by secondary RAS mutations, activation of bypass signalling pathways, or direct resistance-conferring mutations [6,7]. Collectively, these existing therapies demonstrate limitations such as insufficient specificity, high toxicity, and frequent resistance while still posing potential damage to normal cells. This highlights an urgent need for novel, highly effective, and low-toxicity anti-CRC drugs.

Owing to their unique origins and structural characteristics, marine peptides have obvious advantages in fighting CRC. The ocean is an ecosystem with high biodiversity. Organisms adapt to extreme conditions, such as high pressure, salinity, and low temperature, and evolve metabolic pathways that are different from terrestrial life. This process generates peptides with new structures and specific functions [8,9]. These marine-derived peptides directly act on tumour cells, regulate the tumour microenvironment, and participate in immune regulation. They form a rich and unique candidate molecular library for the prevention and treatment of CRC, providing important resources for overcoming the single-target restrictions of traditional drugs [10,11].

Recent studies demonstrate the therapeutic potential of marine peptides. With the continuous exploration of the medicinal potential of marine organisms, the structure and function of marine polypeptides have also been investigated. Ziconotide extracted from conical snails has been approved by the U.S. Food and Drug Administration because of its good targeting activity and low toxicity, setting standards for the safety of peptide drugs [12,13]. Dolastatin 10 has shown positive results in various phase II trials of solid tumours, proving that it has anti-tumour effects [14]. Focusing on CRC, F2, rAj-HRP, and other peptides induce cell apoptosis or inhibit proliferation by regulating the ubiquitinization, cell cycle, and other mechanisms of XIAP, which are promising candidate drugs [15,16,17]. These advances show a clear, gradual research trajectory from basic research on the structure and function of marine peptides to clinical applications for the treatment of tumours.

With varied origins and research bases, marine peptides offer unique merits for CRC therapy. From a physicochemical perspective, their relative small size is conducive to efficient transmembrane transport into tumour cells, which solves the problem of drug delivery, and they are also easy to prepare and modify [18]. Compared with traditional chemotherapy drugs, they have less drug interaction, less accumulation in the liver and kidneys, and a lower incidence of adverse reactions [19]. In terms of mechanism, marine peptides generally come into contact with multiple targets to regulate cell apoptosis, proliferation signals, and the tumour microenvironment, thus overcoming the limitations of single-target agents. Marine peptides are highly selective for cancer cells and can selectively eliminate tumour cells, protect healthy cells, and reduce treatment-related damage [20,21,22]. These comprehensive advantages determine their potential as a new anti-CRC therapy.

In this review, the mechanism of action of marine peptides against colorectal cancer and their potential use in treatment are considered. In what follows, we summarize the sources, structures, and research advancements pertaining to these peptides, describing the transition from basic research to transformation exploration. The goal is to provide a solid theoretical foundation for the future development of marine peptide drugs for the treatment of CRC.

## 2. Source and Structural Characteristics of Marine Peptides

### 2.1. Sources of Marine Peptides

**Figure 1:**
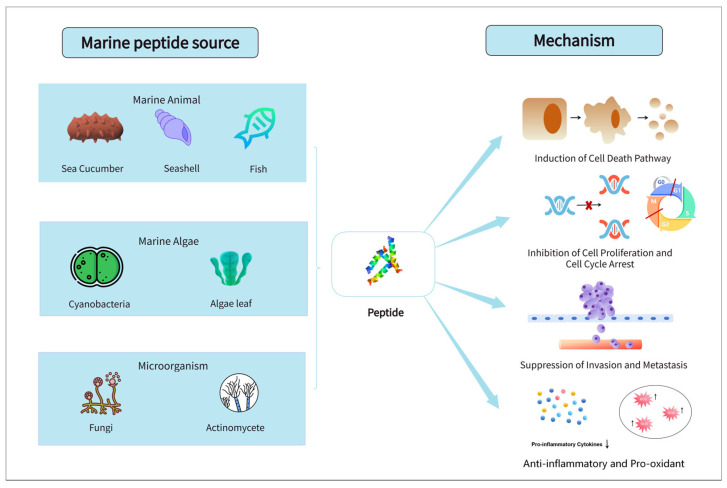
Marine-derived peptides and their mechanisms in inhibiting colorectal cancer.

There are a variety of peptide resources in the marine environment, mainly derived from marine animals, algae, and microorganisms, each of which produces biologically active peptides with unique structures and functional properties [23,24]. Marine animals, including fish, mollusks, crustaceans, echinoderms, sponges, tunicates, and sea cucumbers, are the major sources [25] (Figure 1) (Table 1). Kahalalide F, a cyclic depsipeptide derived from the mollusk *Elysia rufescens*, inhibits colon cancer cell proliferation by disrupting the lysosomal structure of cancer cells [26]. Anti-cancer peptide fractions derived from the bivalve mollusk *Ruditapes decussatus*, particularly the peptide Pep25, exhibit highly selective cytotoxicity against colorectal cancer cell lines by inducing apoptosis, activating autophagy, and inhibiting tumour-proliferative signalling pathways [27]. The sponge produces the cyclic peptides Callyaerin E/G, which are biologically active compounds with cytotoxic effects on tumour cells [28], whereas the tunicate yields the anti-tumour peptide CS5931, which exerts effects via mitochondrial apoptosis and cell cycle arrest [29]. Didemnin B is a cyclic decapeptide initially isolated from the tunicate *Trididemnum solidum*. It exhibits potent antiproliferative activity against colorectal cancer, lung cancer, and other tumour cell lines through multiple mechanisms, including the inhibition of protein synthesis by binding to the eukaryotic elongation factor 1 (eEF1) and the suppression of cyclin expression. Plitidepsin, a derivative of didemnin B from the tunicate *Aplidium albicans*, exerts its anti-tumour therapeutic effects via eukaryotic elongation factor 1 α (eEF1A)-mediated oxidative stress induction and JNK activation [30]. Histidine-rich peptides derive from sea cucumber target epidermal growth factor receptors (EGFR) and their downstream signalling cascade [31]. Furthermore, sea anemones constitute a valuable reservoir of anti-cancer peptides. Actinoporins, a class of proteins and peptides produced by sea anemones, form transmembrane pores in the sphingomyelin-enriched membrane domains of target cells. This process disrupts cellular osmotic balance and induces cell lysis [32]. An actinoporin named Sticholysin I, derived from *Stichodactyla helianthus*, has been conjugated to the anti-colon cancer antigen monoclonal antibody IOR C5. This conjugate effectively eliminates SW948 colorectal cancer cell lines in preclinical models [33]. Colorectal tumour cell membranes are characterized by high levels of sphingomyelin. Thus, the pharmacological activity of actinoporins renders them promising candidate molecules for the treatment of colorectal cancer (CRC) [34].

Green varieties of algae synthesize cyclic peptides. The ring structure is composed of amino acids and fatty acid chains [35]. Red algal peptides often combine with polysaccharides containing monosaccharide residues such as glucose and galactose to form complexes [36]. Brown algal peptides generally function with phenolic groups to regulate apoptosis-related proteins [37]. Peptides derived from cyanobacteria generally contain thiazole rings, which have a strong inhibitory effect on lymphoma cells [38]. Microorganisms, particularly actinomycetes and fungi, are also important sources. Actinomycetes produce proteasome-inhibitory peptides with a γ-lactam-β-lactone bicyclic structure [39], and fungal peptides destroy key signalling pathways to cause anti-tumour activity [40].

### 2.2. Structural Characteristics of Marine Peptides

The structure of a marine peptide is complex and closely related to its biological activity and therapeutic potential for the treatment of colorectal cancer (CRC). Marine peptides mainly occur in a linear or cyclical form, adapted to the harsh marine environment, and perform targeted functions [41]. Linear peptides are composed of straight amino acid chains connected by peptide bonds. Due to their relatively simple sequence, they generally have high tissue permeability, which directly affects the binding affinity with cell targets [42]. Dolastatin 10 is a linear peptide derived from sea hare venom. It inhibits the assembly of microtubules, resulting in an anti-tumour effect [43]. The bivalve linear peptide P6 induces CRC cell apoptosis by regulating the p38 MAPK pathway [44]. For cyclic peptides formed through peptide-bond cyclization or ester-bonds, the metabolic stability is improved due to the reduction in the enzymatic degradation sites of cyclic peptides, thus prolonging the effect in the body [45]. Notable examples include Kahalalide F, a cyclic depsipeptide from green algae, with 13 amino acid rings, and the fatty acid chain is connected by ester bonds, which plays the role of dual structural reinforcement [46]. There are also cryptophycins from cyanobacteria, which have a strong inhibitory effect on microtubules on account of their ring structure [47]. These structural properties account for the effects and resilience of peptides, which have been confirmed by in vitro and in vivo studies.

## 3. Methods for Obtaining and Isolating Marine Peptides

Marine peptide production should be tailored according to the source and structural characteristics—that is, enzyme hydrolysis, direct extraction and purification, fermentation, structural optimization, and other key methods. Each method has its own unique features in terms of efficiency and scalability [48] (Table 2).

### 3.1. Enzymatic Hydrolysis

Enzymatic hydrolysis is the fundamental method of marine peptide generation. It is necessary to achieve precise protein degradation by protease-mediated catalysis, for which the strict regulation of parameters is imperative [49]. When using neutral protease to hydrolyze oyster protein, temperature, pH, and duration should be carefully optimized to improve efficiency [50]. Similarly, regulating parameters in the alcalase-mediated hydrolysis of sea cucumber protein can improve the yield of active peptides [51]. Controlling the molecular weight characteristics and biological activity ensures that peptides are generated effectively. Recent research shows that the enzyme hydrolysis methods used in the production of marine peptides have different effects. Ultrasound-assisted enzymatic lysis has become an efficient method for extracting fish skin protein. In the case of collagen, the yield achieved with the traditional enzymatic method is 20% to 35%. Ultrasound-assisted hydrolysis (20 kHz, 400 to 750 Watt) increases the yield and shortens the processing time by more than 50% [52]. This improvement is due to the structural damage caused by cavitation, which promotes the interaction between enzymes and substrates. When processing the gonadal glands of *Strongylocentrotus nudus*, the optimized papain method yields hydrolyzed antioxidant peptides at 27.96 ± 0.54%; the product demonstrates good antioxidant activity, and the method is markedly superior to the single-enzyme strategy [53]. This sequential method has greater advantages in terms of hydrolysis efficiency and bioactive peptide yield, which meet the requirements of large-scale production of functional marine peptides.

### 3.2. Direct Extraction and Purification

Direct extraction and purification are generally used to separate native marine peptides, and the initial steps should be adapted to different sources. Animal tissues are generally crushed and homogenized to destroy the cell–matrix; then, buffer-assisted extraction is undertaken to maintain the integrity of biological activity and minimize the loss of components [8]. Algal peptides are generally extracted using polar solvents such as methanol, and the initial fractionation is carried out by differential polarity [54]. Crude extracts from microorganisms are recovered from the fermentation liquid by centrifugation and filtration; then, the active ingredients are enriched by concentration [55]. Purification is generally achieved via fine separation chromatography techniques, such as separating callyaerin A from marine sponge *Callyspongia aerizusa* and separating symplostatin A from marine cyanobacteria *Symploca* sp. [56,57]. Multi-stage chromatography is crucial for obtaining high-purity isolates, such as Milnamides A and D from sponges [58] and Bistratamides E–J from tunicates [59]. Recent research has highlighted significant differences in yield and efficiency among extraction methods for marine-derived bioactive compounds. While novel technologies such as microwave-assisted extraction (MW) and supercritical fluid extraction (SFE) exhibit excellent performance in extracting marine lipids and oils, they are generally less suitable for marine peptides. In fact, compared to traditional solvent extraction and enzymatic hydrolysis, SFE often yields inferior results in peptide recovery due to the non-polar nature of supercritical CO_2_, and MW carries a risk of thermally degrading sensitive peptide structures [60]. Therefore, traditional enzymatic hydrolysis, often coupled with advanced ultrafiltration, remains the most reliable and widely adopted approach for efficiently recovering marine-sourced peptides from species such as Atlantic salmon (*Salmo salar*) and *Spirulina platensis* [61]. Nevertheless, SFE and MW serve as valuable complementary processing steps that indirectly benefit peptide characterization. Optimized SFE conditions achieved a defatting yield of 86.75% from *Spirulina platensis*, significantly outperforming conventional Soxhlet extraction (9.18%) and enriching the protein content of the post-extraction solid cake from 69.2 to 74.4 g/100 g. When this protein-enriched cake is subsequently analyzed by LC-QTOF-MS/MS, 14 peptides are identified [62]. These findings suggest that SFE, while not a direct peptide extraction method, indirectly enhances peptide recovery by concentrating proteins in the residual solid. MW pre-treatment also significantly releases specific protein fractions from *Chlorella vulgaris*, facilitating subsequent enzymatic hydrolysis and peptide formation, though its contribution to overall protein extraction yield is not statistically significant, and the risk of thermal degradation of heat-sensitive peptides should be noted [63].

### 3.3. Fermentation

Fermentation has emerged as an effective and sustainable approach for the large-scale production of bioactive marine peptides. By utilizing microbial biosynthesis, efficient peptide accumulation can be achieved while valorizing marine biomass. An illustrative case is the microbial fermentation of marine processing by-products (such as fish skin, viscera, or tuna frame proteins) using selected strains like *Bacillus subtilis* or lactic acid bacteria. This bioconversion process not only facilitates the cost-effective and environmentally friendly mass production of functional marine peptides but also resolves the ecological issues associated with marine waste disposal [64]. Recent surveys show that the optimized fermentation strategies of marine peptide production is effective. In red seaweed (*Pyropia* spp.), solid-state fermentation (SSF) of the complex lactic acid bacteria (LAB) consortium improves the soluble protein content and peptide diversity, producing 58 bioactive peptides, which are superior to single-strain fermentation in terms of yield and function [65]. In addition, oysters (*Crassostrea angulata*) were fermented underwater with *Lactobacillus casei* to obtain peptides mainly composed of low-molecular-weight fractions (<1000 Da). These peptides have strong gastrointestinal stability and immunomodulation. The fermentation process will not use harsh chemicals, which minimizes waste [66]. The optimized fermentation method improves the output of the target peptide and the retention of biological activity and overcomes the many limitations of traditional batch fermentation, reflecting its advantages in sustainable marine peptide production.

### 3.4. Structural Optimization

Structural optimization refers to the use of molecular modification to expand the biological activity of natural peptides. Taking the new peptide identified in the hydrolysis of *Ostrea rivularis Gould* protein as the target, reasonable optimization with molecular docking was achieved. Researchers used Trp to replace the N-terminal Gly of GGYGIF or designed alternate simple and hydrophobic amino acid sequences to produce the derivative WGWGW, whose xanthine oxidase-inhibitory activity is more than twofold higher than that of the original peptide [67]. Structural optimization of the marine sponge-derived cyclic peptide stylissatin A (SA) has been primarily achieved through the specific modification of its tyrosine residue. Research indicates that the protection of the tyrosine hydroxyl group—specifically by introducing *tert*-butyl, propargyl, methyl, or benzyl groups to form the corresponding ethers—markedly enhances the nitric oxide (NO)-reducing activity of SA derivatives. Consequently, the optimized analogue exhibits significantly more potent anti-inflammatory activity (EC_50_ = 12 μM) compared to the natural SA (EC_50_ = 73 μM) [68]. These modifications exemplify how rational design, guided by structure–activity relationships and molecular docking, substantially elevates peptide functionality and identifies avenues for the production of high-efficacy marine peptides.

## 4. Mechanistic Analysis of Marine Peptides

### 4.1. Induction of Cell Death Pathways

A core mechanism by which marine peptides combat colorectal cancer is the regulation of cell death. They directly trigger cancer cell death through diverse and targeted approaches, including the activation of apoptosis pathways and the induction of cytotoxic damage. To activate apoptosis, marine peptides primarily target the mitochondrial-dependent intrinsic pathway and block key signalling cascades. rAj-HRP from the marine mollusk *Babylonia areolata* activates the mitochondrial apoptosis pathway by upregulating the pro-apoptotic protein BAX and catalyzing PARP degradation. It also targets the EGFR and its downstream PI3K/AKT and Ras/MAPK signalling axes, creating a synergistic effect of apoptosis activation and signal inhibition with an IC_50_ as low as 5.21 μM in HCT116 cells [16]. The P6 peptide isolated from the bivalve *Arca inflata* promotes ROS generation, which induces calcium overload, disrupts mitochondrial membrane potential, and activates the p38 MAPK pathway to induce apoptosis in HT-29 and DLD-1 cells. A 30 mg/kg dose of P6 achieved a tumour inhibition rate of 72.66% in a nude mouse xenograft model [44,69]. Actinomycin V, produced by the marine actinomycete *Streptomyces* sp., disrupts the mitochondrial membrane, potentially leading to the release of cytochrome c into the cytoplasm. This subsequently activates cleaved caspase-9/3 and PARP while inhibiting the pro-survival signals of the PI3K/AKT pathway, showing potent cytotoxic activity on HCT116 and HT-29 cells [70]. Trichodermamide B, a metabolite from the marine fungus *Penicillium janthinellum* SH0301, specifically blocks the JAK/STAT3 pathway by inhibiting STAT3 phosphorylation at the Y705 site and downregulating downstream anti-apoptotic proteins, thereby inducing apoptosis in HCT116 cells [44].

In terms of cytotoxic damage, marine peptides cause cell death by directly disrupting structural integrity or interfering with cytoskeletal function. The HVLSRAPR peptide from *Spirulina platensis* exhibits selective cytotoxicity against HT-29 cells with an IC_50_ of 99.88 μg/mL, while its toxicity to normal liver cells is negligible. Dolastatin 10 from the sea hare *Dolabella auricularia* specifically binds to tubulin, inhibiting microtubule polymerization and disrupting spindle assembly, which blocks cell division and triggers apoptosis [71]. Desmethoxymajusculamide C (DMMC), which is produced by the cyanobacterium *Lyngbya majuscule*, targets the cytoskeleton, disrupting the microfilament network and cell morphology of HCT116 cells at a low IC_50_ of 20 nM, leading to apoptosis [72]. Marine peptides mediate colorectal cancer cell death via the targeted activation of apoptotic pathways and the induction of cytotoxic damage through cell structure and cytoskeletal disruption, demonstrating significant potential in the regulatory process.

### 4.2. Inhibition of Cell Proliferation and Cell Cycle Arrest

Another major mechanism through which marine peptides block colorectal cancer progression is the inhibition of cell proliferation and the regulation of the cell cycle. They intervene in cell cycle checkpoints and proliferative signalling pathways to halt the unlimited division of cancer cells. The orderly progression of the cell cycle depends on the regulation of cyclin-dependent kinase (CDK) and cyclin complexes. Marine peptides can interfere with the binding of these two components or directly inhibit CDK activity, causing cells to arrest at a specific phase [73]. Based on the arrest phase, these mechanisms can be divided into G0/G1 and G2/M phase arrest. Thiocoraline, which is produced by the marine actinomycete *Micromonospora marina*, specifically binds to the active site of DNA polymerase α, inhibiting its catalytic function and impeding the initiation of DNA replication in LoVo and SW620 cells, ultimately leading to G1 phase arrest [74]. Ohmyungsamycin A, which is secreted by a marine Streptomyces species, reduces the activity of key molecules required for cell cycle progression by modulating the Skp2-p27 axis and MCM4 protein expression, inducing cells to remain in the G0/G1 phase and blocking cancer cell division from its initiation [75]. Asperphenin A, a lipopeptide benzophenone derivative secreted by the marine fungus *Aspergillus* sp., specifically acts on RKO cells by interfering with the function of mitosis-related proteins, inducing G2/M phase arrest, and preventing cells from entering the division stage. In terms of inhibiting proliferative signalling pathways, marine peptides mainly target core pathways closely associated with colorectal cancer development, such as PI3K-Akt-mTOR. By inhibiting the phosphorylation of key proteins in these pathways and blocking the signalling cascade, they suppress the transmission of proliferation signals at the source [76]. By targeting distinct regulatory nodes in the cell cycle and core proliferative signalling cascades, marine peptides present reliable inhibitory effects against the uncontrolled growth of cancer cells.

### 4.3. Suppression of Invasion and Metastasis

A key mechanism by which marine peptides curb colorectal cancer progression and improve prognosis is the suppression of invasion and metastasis. They achieve this by targeting core steps in tumour metastasis, creating a comprehensive blockade of the metastatic process [77]. The epithelial–mesenchymal transition (EMT), the initial step in metastasis, endows cancer cells with migratory and invasive capabilities. Marine peptides effectively block the EMT by regulating EMT-related transcription factors and markers. Nobilamide I, produced by the marine actinomycete *Saccharomonospora* sp., downregulates EMT transcription factors like Snail and Slug. It also inhibits the activity of matrix metalloproteinases (MMPs) 2/9 and upregulates the expression of their inhibitor, TIMP2, significantly inhibiting the migration of Caco2 cells [78]. Androsamide, from the actinomycete *Nocardiopsis* sp., blocks the EMT in Caco-2 and HCT116 cells by downregulating Snail, Slug, and the mesenchymal marker N-cadherin, thereby reducing the invasive capacity of cancer cells [79]. The degradation of the extracellular matrix (ECM) is a necessary step for tumour cell invasion and metastasis and is primarily mediated by proteases such as matrix metalloproteinases (MMPs) [80]. Tasiamides from the cyanobacterial genus *Symploca* sp. exhibit an IC_50_ of 3.47 μg/mL in LoVo cells, and their derivatives specifically inhibit the enzyme cathepsin D. By inhibiting cathepsin D, these peptides reduce the degradation of the extracellular matrix (ECM) by tumour cells and suppress cancer cell invasion [81].

Furthermore, marine peptides curb metastasis by interfering with cytoskeletal function and inhibiting angiogenesis. Hemiasterlin, a peptide isolated from the sponge *Auletta* sp., induces microtubule depolymerization and exerts anti-mitotic effects against tumour cells [17]. The CS5931 peptide from the sea squirt *Ciona savignyi* inhibits the expression of angiogenesis-related factors by disrupting the cell membrane structure. It is not only highly toxic to HCT-8 cells but also inhibits their proliferation and migration at low concentrations, blocking the formation of metastatic foci from the perspective of vascular supply [82]. Different marine peptides exert these distinct anti-metastatic mechanisms by targeting individual key nodes of the metastatic process, such as EMT initiation, extracellular matrix degradation, cancer cell migration, and tumour angiogenesis, reflecting the diversity and specificity of marine peptides in inhibiting colorectal cancer metastasis.

### 4.4. Anti-Inflammatory and Pro-Oxidant Properties

The anti-inflammatory and pro-oxidant properties of marine peptides are important mechanisms for improving the tumour microenvironment and indirectly inhibiting colorectal cancer progression. These mechanisms depend on regulating the balance of inflammatory factors and controlling oxidative stress levels, thereby eliminating the pro-carcinogenic characteristics of the tumour microenvironment [83].

In terms of anti-inflammatory effects, marine peptides primarily work by inhibiting the expression of pro-inflammatory factors and blocking inflammatory signalling pathways. The H-TL1 polypeptide secreted by the sea snake *Hydrophis cyanocinctus* acts as a TNF antagonist, competitively binding to the TNF receptor. This blocks the activation of the NF-κB inflammatory signalling pathway, reducing the expression of pro-inflammatory factors like IL-1β and COX-2 and inhibiting inflammation-mediated tumour proliferation and invasion [84]. Grassypeptolides, from the marine cyanobacterium *Lyngbya confervoides*, not only inhibit HT-29 colorectal cancer cells but also specifically inhibit dipeptidyl peptidase 8 (DPP8). DPP8 regulates T-cell activation-related inflammation, reducing the secretion of the pro-inflammatory cytokine IL-2 and balancing the state of abnormally activated T-cells in the tumour microenvironment, achieving a dual regulation of anti-inflammation and immune preservation [85].

In terms of pro-oxidant regulation, specific marine peptides exert their anti-cancer effects by elevating reactive oxygen species (ROS) levels beyond the toxic threshold in cancer cells, thereby inducing severe oxidative damage. For example, Pardaxin, a well-characterized peptide isolated from the marine flatfish *Pardachirus marmoratus*, has been shown to induce massive intracellular ROS accumulation, which subsequently triggers oxidative DNA damage and caspase-dependent apoptosis in human cancer cells [86]. Similarly, the marine-derived antimicrobial peptide Tilapia piscidin 4 (TP4) effectively elevates ROS levels within cancer cells, leading to severe oxidative stress, mitochondrial dysfunction, and dose-dependent cytotoxicity against various malignant cell lines [87]. By participating in regulation, marine peptides can break the vicious cycle of inflammation and cancer and create favourable conditions for the treatment of colorectal cancer.

## 5. In Vivo and In Vitro Anti-Colorectal Cancer Efficacy of Marine Peptides

In vitro investigations serve as a foundational screening tool for marine peptides’ anti-CRC efficacy, allowing for the precise assessment of antiproliferative, pro-apoptotic, and anti-metastatic effects under controlled conditions and the identification of promising candidates for mechanistic elucidation [88]. There are a wide variety of marine peptides with strong activity [89]. Halipeptin D is a cyclic sponge-derived peptide with an IC_50_ of 7 nM for HCT116 cell line. Largazole, from cyanobacteria *Symploca* sp., specifically binds to the catalytic domain of HDAC1 to inhibit histone deacetylase (HDAC) and induce HCT116 cell apoptosis at the nanomolar level [90]. Rakicidin C, derived from *Streptomyces* sp. GKU 220, inhibits the invasion of 26-L5 cells in the mouse colon at 1.25 μM [91]. Fish-derived peptides, such as *Rainbow trout* skin hydrolysates, inhibit the survival of HCT116 [92], and the by-products of flathead fish achieved 91% growth inhibition in HT-29 cells [93]. These studies show how cyclization and other structural characteristics enhance biological activity through stable interaction with cellular targets, which provides mechanical insights into the relationship between peptide structure and CRC inhibition. To better explain the in vitro efficacy of marine peptides, we compiled the molecular features of CRC cell lines in Supplementary Table S1, using Cellosaurus as the primary reference. This is biologically meaningful because KRAS and BRAF mutations influence the EGFR/RAS/MAPK signalling axis, PIK3CA mutations activate the PI3K/AKT survival pathway, APC/CTNNB1 alterations remodel Wnt-driven proliferation and cytoskeletal organization, TP53 defects affect mitochondrial apoptosis and DNA damage responses, SMAD4 loss facilitates metastatic behaviour, and MSI-H/dMMR status alters replication stress and apoptotic thresholds. Consequently, marine peptides targeting these pathways may show differential potency across CRC cell lines with distinct genetic backgrounds. For example, the marine peptide P6 is more potent in KRAS/PIK3CA-mutant DLD-1 (IC_50_ = 2.14 μM) than in BRAF-mutant HT-29 (IC_50_ = 4.43 μM), suggesting sensitivity to KRAS/PIK3CA signalling. Thiocoraline differs in activity between SW620 (IC_50_ = 15 nM) and LoVo (IC_50_ = 500 nM), implying background-dependent targeting of DNA replication. However, current studies have not yet directly compared isogenic KRAS, BRAF, or PIK3CA-mutant versus wild-type CRC cell lines; thus, the relationship between peptide potency and specific mutations remains correlative, not causative. Future work should evaluate marine peptides in isogenic CRC cell models carrying defined KRAS, NRAS, BRAF, PIK3CA, APC, TP53, or mismatch repair alterations to determine whether particular oncogenic mutations modulate peptide sensitivity or resistance.

Supplementing in vitro findings, the in vivo models further demonstrate that marine peptides are effective against CRC in a complex physiological environment, taking into account the immune response, bioavailability, and toxicity to better predict clinical results [94]. The immunocompetent model is suitable for immunomodulatory peptides such as ohmyungsamycin A from marine actinomycete, which achieved 52.1% tumour suppression in HCT116 xenograft mice without immune-related damage [74]. Immunodeficient xenograft models, ideal for direct anti-tumour assessment, demonstrate efficacy in peptides such as rAj-HRP, inhibiting HCT116 tumour volume by nearly 60% at 200 μg/kg in nude mice with minimal toxicity [16], and P6 from *Arca inflata*, yielding over 70% inhibition in HT-29 xenografts [44]. Specialized alternative models, such as zebrafish xenograft models, provide efficient in vivo preliminary screening tools for peptides by taking advantage of transparent embryos and short experimental cycles [95]. Cyclic dipeptides derived from *Escherichia* sp. reduced tumour volume by over 60% in the HT-29 zebrafish model, with no observed embryotoxicity, confirming their favourable preclinical characteristics [96].To provide a more transparent comparison of in vivo efficacy, the experimental parameters, including animal model, CRC cell line, dose, route of administration, dosing frequency, treatment duration, tumour inhibition rate, and toxicity observations have been summarized in Supplementary Table S2.

In general, these in vivo verifications confirm that the structural optimization of marine peptides is transformed into effective CRC inhibition, highlighting the low toxicity characteristics and laying the foundation for overcoming the conversion bottleneck through innovative technologies.

## 6. Application of Marine Peptides in the Treatment of Colorectal Cancer: Breakthroughs and Technological Innovations

### 6.1. Translation Challenges: Key Constraints for Application of Marine Peptides

The clinical adoption of marine peptides in the treatment of colorectal cancer (CRC) is hindered by the challenges of interrelated challenges in CRC-specific efficacy, pharmacokinetics, scalable production, and safety evaluation, which hinders their transition from preclinical research to clinical application. There is a lack of optimized drug-like properties adapted to CRC-specific conditions, a production system suitable for the clinical needs of CRC, and safety mechanism evaluation consistent with CRC pathophysiology [97].

A primary bottleneck lies in the poor optimization of marine peptides’ drug-like properties for CRC’s unique physiological microenvironment [98]. Most linear marine peptides are highly susceptible to degradation by CRC-overexpressed proteases such as matrix metalloproteinases and aminopeptidases in the tumour stroma and bloodstream [99]. For instance, sea cucumber-derived peptides exhibit substantial degradation within 1 h in CRC xenograft mice, while algae/shellfish-derived peptides have short in vivo half-lives, failing to maintain effective concentrations in CRC tumour tissues with dense stromal barriers [83,100]. These problems are important because many reported marine peptides are of medium molecular size. They are larger and less permeable than small-molecule drugs but lack the long circulation and target-recognition properties of antibodies [101]. Consequently, their oral bioavailability is often limited by gastrointestinal peptidases, poor epithelial permeability, and first-pass clearance, whereas systemic administration is still constrained by serum proteolysis, renal filtration, and rapid metabolic inactivation [102]. Hydrophilic peptides cannot penetrate the collagen-rich CRC stroma, while hydrophobic peptides aggregate in aqueous solutions [103]. In addition, medium-sized peptides generally do not freely diffuse across tumour-cell membranes and often require endocytosis, transporter-mediated uptake, or cell-penetrating motifs. However, endosomal or lysosomal trapping may prevent their access to intracellular targets such as mitochondria and cytoskeletal proteins [104]. Dense extracellular matrix, abnormal vasculature, and high interstitial pressure in CRC further reduce tumour-cell accessibility. Potential neurotoxicity also needs explicit assessment, especially for marine peptides affecting ion channels, neuronal membranes, or microtubules, because off-target effects on peripheral or enteric neurons may cause neuropathy or gastrointestinal dysmotility [105]. This phenomenon is commonly observed in CRC patients with tumour-induced gut barrier dysfunction and further lowers peptide bioavailability [106]. Critically, existing peptide delivery systems rely on non-specific tumour microenvironmental cues, such as low pH or high glutathione, rather than CRC-specific biomarkers, such as carcinoembryonic antigen, microsatellite instability, and HER2 [107]. This mismatch results in poor tumour enrichment and an increased risk of damage to normal colonic epithelia, which is particularly concerning for CRC patients with pre-existing intestinal inflammation or polyposis [108].

Also, the production system fails to meet the clinical scalability requirements of CRC. Natural extraction is limited by the scarcity of marine resources and complex workflows [109]. Isolated Dolastatin 10 is a candidate drug for the treatment of CRC. Its isolation requires the processing of a large number of raw materials, followed by repeated chromatography. Due to the high incidence of CRC and the high demand for large-scale clinical supplies, this practice is unsustainable [14]. Thus, chemical syntheses such as solid-phase peptide synthesis include obstacles to CRC-targeted peptides [110]. Long-chain peptides modified with CRC-specific ligands are difficult to synthesize on a large scale and require multiple HPLC purifications, which increase the fluctuations in cost and yield, affecting phase I/II CRC trials [111].

Compounding these barriers are insufficient CRC-aligned safety assessments and mechanistic gaps [112]. Toxicological studies for most marine peptides are limited to in vitro cytotoxicity assays, lacking evaluations of systemic safety in CRC-relevant contexts [113]. This limitation is largely attributed to the lengthy ethical approval process for in vivo studies, the high costs of animal modelling and clinical patient recruitment, and the substantial time investment. Although most marine peptides show no toxicity to normal cells in vitro, the complex metabolic environment in vivo can amplify their non-specific toxicity [99]. Adverse effects tend to accumulate preferentially in metabolic organs such as the colon, liver, and kidneys [114]. For instance, the broad-spectrum anti-tumour peptide Didemnin B was terminated in phase II clinical trials because of intestinal toxicities such as nausea and vomiting at elevated doses and a lack of efficacy in patients with advanced cancer. The peptide Plitidepsin, despite advancing to phase III trials, causes adverse reactions, including fatigue and nausea, at high doses. Therefore, the clinical safety of marine peptides must be validated through in vivo experiments to determine dose thresholds, organ-specific toxicity, and long-term tolerance so as to balance anti-cancer activity with the protection of normal tissues [115]. Additionally, marine peptides’ non-endogenous sequences carry immunogenicity risks, which are particularly critical for CRC patients undergoing chemotherapy or immunotherapy, for example, via anti-PD-1 agents, as immune activation could exacerbate adverse reactions or compromise treatment efficacy [116,117]. Mechanistically, although existing studies have confirmed that some marine peptides target partial CRC-associated signalling pathways, most investigations still have mechanistic research limitations. They lack the systematic identification and in-depth analysis of key CRC-specific molecular targets, such as the Wnt/β-catenin pathway, alongside their complete upstream and downstream regulatory networks [118]. No subtype-specific mechanistic verification and exploration have been carried out for distinct CRC molecular subtypes, including MSI-H, MSS, and BRAF-mutant subtypes [119]. This gap prohibits the establishment of a clear correlation between the modes of action of marine peptides and the genetic and pathological characteristics of various CRC subtypes, which further slows the clinical translation of marine peptides for CRC therapy [11,120].

### 6.2. Technological Innovations: Tailored Strategies to Improve Translational Efficacy

**Figure 2:**
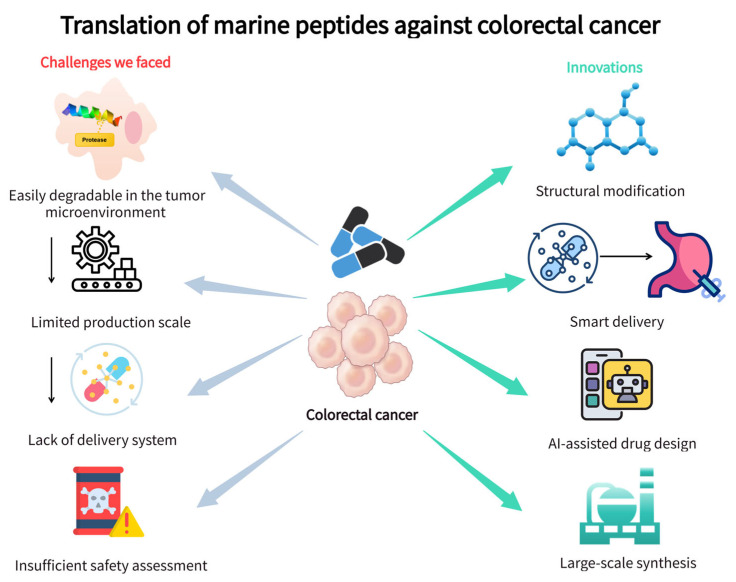
Clinical application challenges and innovative solutions of marine peptides in colorectal cancer treatment.

Technological innovation tailored to the unique pathophysiology of CRC is pivotal to overcoming these challenges. The main avenues of progress in this regard include the optimization of drugs adapted to CRC, the scalable production of cancer-specific peptides, and the acceleration of the transformation of the latest technologies (Figure 2).

#### 6.2.1. Optimization of Drug-like Properties for CRC

The structural transformation and intelligent delivery system are designed to solve the protease-rich tumour microenvironment and matrix barrier of CRC. Cyclization enhances the resistance of matrix metalloproteinase and aminopeptidase overexpressed by CRC and is expected to prolong the in vivo half-life of marine peptides in xenografts [121,122]. In addition, a colorectal cancer biomarker guidance system was developed. The carcinoembryonic antigen (CEA)-targeted transport vector—that is, anti-CEA monoclonal antibodies bound to cell osmotic peptides—encapsulates peptides with customized pharmacokinetic characteristics and achieves targeted accumulation in the CEA-positive CRC model [123]. Meanwhile, pH-responsive nanoparticles respond to the pH of the microenvironment of CRC tumours, which control peptide release, thus reducing the off-target toxicity to normal colon tissue [124]. PEGylated nanoparticles reduce the clearance of the reticuloendothelial system, which is an important improvement for CRC patients who experience impaired drug circulation due to liver metastases [125,126]. To overcome pharmacokinetic and cellular delivery barriers in CRC therapy, future peptide optimization should combine structural stabilization with tumour-targeted delivery strategies. Strategies such as cyclization, D-amino acid substitution, terminal amidation, lipidation, and PEGylation enhance resistance to proteolytic degradation, reduce renal clearance, and extend systemic circulation, though their impact on target affinity and toxicity necessitates validation. Nanocarriers, liposomes, polymeric micelles, and hydrogel depots may protect peptides from enzymatic degradation and enhance accumulation in CRC lesions. To improve membrane transport and intracellular availability, peptides can be conjugated with tumour-activated cell-penetrating sequences, pH-sensitive linkers, or endosomolytic motifs that promote endosomal escape [101,102]. CRC-specific ligands targeting CEA, epithelial cell adhesion molecule (EpCAM) and integrins may further increase tumour-cell accessibility and reduce normal-tissue exposure. Optimized peptides should be evaluated via serum stability assays, neurotoxicity screens, and in vivo pharmacokinetic–pharmacodynamic studies [104,105].

#### 6.2.2. Scalable Production of CRC-Specific Marine Peptides

Synthesis technology has been improved to meet the requirements of clinical scale, mainly through research on efficiency, purity control, and compatibility with the characteristics of CRC active peptides. The combination of automatic solid-phase peptide synthesis (SPPS) and microwave assistance is still the main way of synthesizing bioactive peptides, which solves problems such as peptide-chain aggregation and steric hindrance [127,128]. Enzyme synthesis complements the chemical method of hydrolyzing the marine protein substrate at a specific location with food-grade protease. The continuous membrane reactor system recovers enzymes, reduces reagent consumption, and shortens the production cycle, thus further improving the enzyme process [129,130]. This strategy is suitable for producing peptides with CRC-inhibitory activity and minimizing the impact on the environment [131]. Purification technology and synthesis workflow are carried out at the same time to ensure clinical compliance [132,133]. Ultrafiltration based on molecular weight enriches biologically active peptides; then, reverse-phase high-performance liquid chromatography (RP-HPLC) is used for fine purification [134,135]. These integrated processes overcome the limitations of natural extraction while ensuring consistent batch quality, laying a solid foundation for the scalable supply of CRC-specific marine peptides and accelerating their translational progress [136,137].

#### 6.2.3. AI-Driven Acceleration of CRC-Targeted Translational Research

Looking ahead, accelerating the translational process of marine peptides for colorectal cancer (CRC) therapy will largely rely on the deep integration of artificial intelligence (AI) tailored to CRC’s unique molecular subtypes and tumour microenvironment (TME). Deep learning technologies are now empowering CRC-specific mechanistic elucidation and high-efficiency candidate peptide screening. Structural prediction tools such as AlphaFold and trRosetta accurately model the three-dimensional conformations of peptides and their binding modes with CRC core targets, which can be further validated through molecular docking and molecular dynamics simulations to clarify interaction mechanisms [138,139].

Beyond structural prediction, AI algorithms integrate tumour microenvironment (TME) characteristics and peptide physicochemical properties with multi-dimensional features to establish subtype-selective screening models [140]. For instance, generative adversarial networks (GANs) effectively mitigate the limitations of limited and imbalanced anti-cancer peptide (ACP) datasets by generating bioactive peptide samples, enabling high-throughput screening of peptides with enhanced efficacy against refractory cancer subtypes [141]. Additionally, explainable AI (XAI) tools such as SHAP and Grad-CAM decode key peptide features driving CRC-specific activity, while multimodal AI frameworks combine pathological image analysis with peptide design to align candidates with clinical pathological features [142]. These technologies have been verified and applied for de novo sequence generation and preclinical efficacy prediction in CRC peptide drug design. By improving target specificity, subtype adaptability, and translational reliability, the technologies significantly accelerate the translation of marine peptides from discovery to clinical application.

## 7. Conclusions

The high incidence and mortality rate of colorectal cancer (CRC), coupled with the lack of existing treatment methods, has necessitated the emergence of new, low-toxicity, and efficient prevention and treatment programmes. Relying on the unique evolutionary advantages of marine ecosystems, marine active peptides show structural diversity and mechanical characteristics. The multi-dimensional regulation of tumour progression to improve the efficacy of conventional treatment represents a very promising approach to CRC prevention and treatment, providing a differentiated means of overcoming current therapeutic challenges. However, there are still obstacles to the clinical transformation of marine peptides, such as sub-excellent drug-like characteristics, restrictions on scalable production, and insufficient system safety evaluation in-depth mechanism clarification. Future progress will rely on the integration of a variety of technologies, such as artificial intelligence-assisted design and nanodelivery systems, and strengthened interdisciplinary cooperation. It is crucial to establish a correlated system linking a marine peptide library with CRC clinical sample banks and to develop unified standards for quality control and formulation processes. Concurrently, further research into formulation stability and human metabolic patterns is needed. As these translational frameworks are gradually refined, marine peptides are poised to be developed into novel anti-CRC drugs that balance efficacy with safety. This will enrich the portfolio of precision treatment options and provide significant support for alleviating the global disease burden and improving quality of life for patients.

## Figures and Tables

**Table 1:** Representative marine peptides with anti-colorectal cancer activity.

Biological Source	Peptide	Species	Structure	Efficacy Against Colorectal Cancer (CRC)	Anti-CRC Mechanism
Marine Animals	rAj-HRP	*Apostichopus japonicus*	Linear peptide	HCT116 IC_50_ 5.21 μM	Upregulate BAX and PARP degradation; suppress EGFR/PI3K/AKT and Ras/MAPK signalling
	P6	*Arca inflata*	Linear peptide	HT-29 IC_50_ 4.43 μM and DLD-1 IC_50_ 2.14 μM; 72.66% tumour inhibition in vivo at 30 mg/kg	Induce ROS burst and calcium influx; activate p38 MAPK and mitochondrial apoptosis pathway
	Dolastatin 10	*Dolabella auricularia*	Linear pentapeptide	Potent cytotoxicity at sub-nanomolar concentrations against multiple CRC lines	Bind tubulin at the vinca domain and inhibit microtubule polymerization
	CS5931	*Ciona savignyi*	Linear peptide	High toxicity against HCT-8 and HCT116 cells	Induce G2/M phase arrest; mitochondrial pathway apoptosis; destruction of cell membrane morphology
	Halipeptin D	*Leiosella* cf. *arenifibrosa*	Cyclic peptide	HCT116 IC_50_ 7 nM	Unreported
	HVLSRAPR	*Spirulina platensis*	Linear octapeptide	HT-29 IC_50_ 99.88 μg/mL	Unreported
	Didemnin B	*Trididemnum solidum*	Cyclic depsipeptide	Inhibit colorectal cancer cells at 10 nM; completed phase II clinical trials	Inhibit protein synthesis; induce mitochondrial apoptosis; interfere with the G1/G2 phase of the cell cycle
	Plitidepsin	*Aplidium albicans*	Cyclic peptide	Completely inhibit colorectal cancer cells at 10 nM; already entered phase III clinical trials	Target eEF1A2; induce apoptosis; inhibit cell cycle progression
	Kahalalide F	*Elysia rufescens*	Cyclic depsipeptide	IC_50_ 0.16–0.29 µM for colorectal cancer cells; has entered phase I clinical trials	Cytoplasmic swelling and vacuolization; damage to mitochondria and lysosomal functions; inducing cell necrosis
	Rainbow trout skin hydrolysates	*Oncorhynchus mykiss*	Peptide segment	HCT116116: <3 kDa peptide IC_50_ = 249.5 μg/mL; <3 kDa peptide IC_50_ = 727.4 μg/mL; >30 kDa peptide IC_50_ = 1446.0 μg/mL	Antioxidant activity and cytotoxicity; inducing apoptosis through high mobility
	Flathead fish by-products peptides	*Platycephalus fuscus*	Linear peptide	Inhibit 91.04% HT-29 at 0.005 mg/mL	Non-selective cytotoxicity; reducing oxidative stress by clearing free radicals
Marine Microorganisms	Actinomycin V	*Streptomyces* sp.	Cyclic peptide	HCT116 IC_50_ 2.85 ± 0.10 nM; HT-29 IC_50_ 6.38 ± 0.46 nM; SW620 IC_50_ 6.43 ± 0.16 nM; SW480 IC_50_ 8.65 ± 0.31 nM	Disrupt mitochondrial membrane potential and activate caspase-9/3; inhibit PI3K/AKT signalling pathway
	Ohmyungsamycin A	*Streptomyces* sp. SNJ042	Cyclic peptide	HCT116 IC_50_ 7.61 μM; 52.1% tumour suppression in vivo	Regulate the Skp2-p27 axis to induce G0/G1 arrest; downregulate MCM4 to disrupt DNA replication; activate caspase-3/7/8 and PARP cleavage to induce apoptosis
	Thiocoraline	*Micromonospora marina*	Cyclic thiodepsipeptide	LoVo IC_50_ 500 nM; SW620 IC_50_ 15 nM	Combine with inhibition of primer extension at the DNA polymerase α active site to block DNA replication
	Nobilamide I	*Saccharomonospora* sp. CNQ-490	Cyclic depsipeptide	60% migration inhibition in Caco2 cells	Downregulate Snail/Slug; inhibit MMP-2/9; upregulate TIMP2
	Androsamide	*Nocardiopsis* sp.CNT-189	Cyclotetrapeptide	Caco2 IC_50_ 13 μM. Migration inhibition at 1.3 μM	Inhibit EMT by downregulating Snail, Slug, and N-cadherin; reduce the expression of genes related to cell motility
	Rakicidin C	*Streptomyces* sp.	Cyclic depsipeptide	Inhibit invasion of 26-L5 colon cells at 1.25 μM	Anti-tumour invasion
	Trichodermamide B	*Penicillium janthinellum* SH0301	Cyclic peptide	HCT116 IC_50_ 0.12 μM, 65% tumour inhibition rate at 20 mg/kg	Inhibit STAT3 phosphorylation at Y705 and block JAK/STAT3 pathway
	Asperphenin A	*Aspergillus* sp.	Lipopeptide benzophenone	Block RKO cells in the G2/M phase (increase the proportion of cells in this phase to 43.94% at 1.25 μM); inhibit tumour growth by 38.9%/68.7% at 4/8 mg/kg	Inhibit microtubule polymerization; promote ROS production; affect the function of mitosis-related proteins
	Cyclo(L-Pro-L-Phe) (DKP-3)	*Exiguobacterium acetylicum* S01	Cyclodipeptide	HT-29 IC_50_ 85.19 μM, 68% tumour inhibition in vivo	Activate mitochondria-mediated apoptotic pathway
Marine Algae and Cyanobacteria	Desmethoxymajusculamide C (DMMC)	*Lyngbya majuscula*	Cyclic depsipeptide	HCT116 IC_50_ 20 nM; administration at 0.62 mg/kg results in 60% tumour proliferation in vivo	Disrupt actin microfilament network; induce changes in cell morphology and formation of binucleated cells
	Grassypeptolides A–C	*Lyngbya confervoides*	Cyclic depsipeptide	HT29 IC_50_: A 1.22 μM, B 4.97 μM, C 76.7 nM	Inhibit DPP8; regulate T-cell activation-related inflammation; induce G1/G2/M phase arrest
	Tasiamide	*Symploca* sp. NIH304	Acyclic peptide	LoVo IC_50_ 3.47 μg/mL	Cathepsin D as a potential anti-cancer target
	Largazole	*Symploca* sp.	Cyclic depsipeptide	HCT116 apoptosis: ≥10 nM induces cell cycle arrest and apoptosis; inhibits tumour growth at 5 mg/kg in vivo	Bind HDAC1 catalytic domain and inhibit histone deacetylase; regulate the cell cycle; induce apoptosis
	Cryptophycins	*Nostoc* sp. strain ATCC 53789	Macrolactone peptide	High anti-tumour activity in preclinical trials	Interfere with microtubule dynamic; inhibit vinblastine binding; prevent mitotic spindle formation; lead to cell cycle arrest and apoptosis
	Tilapia piscidin 4 (TP4)	*Oreochromis niloticus*	Linear peptide	HT-29 IC_50_ 15 μg/mL	Elevate intracellular ROS leading to mitochondrial dysfunction and apoptosis

**Table 2:** Preparation and separation technology of marine peptides.

Method	Representative Peptide	Source Organism	Key Procedure	Advantages
Enzymatic hydrolysis	Antioxidant peptides	*Strongylocentrotus nudus*	Papain hydrolysis (48.83 °C, pH 6.92, enzyme/substrate 3143 U/g, substrate 83.5 g/L, 3 h); then, inactivate the enzyme at 100 °C for 10 min, and finally, separate it using 10 kDa ultrafiltration	High specificity; gentle conditions preserve nutrients; products contain all essential amino acids, with no side effects
Ultrasound-assisted Enzymatic hydrolysis	Acid-soluble collagen	*Lateolabrax japonicus*	20 kHz ultrasound treatment (80% amplitude, 20/20 s pulse, 4 °C) followed by 0.5 M acetic acid extraction, centrifugation to separate the supernatant, and SDS-PAGE analysis	Reduce processing time to 1.5 h; improve extraction rate; maintain the integrity of α1/α2/β chain structures
Direct extraction and chromatographic purification	Callyaerins A	*Callyspongia aerizusa*	Organic solvent extraction followed by multi-stage HPLC	High-purity isolates suitable for structural elucidation
Supercritical fluid extraction (Indirect)	Fourteen peptides	*Spirulina platensis*	Use Jasco supercritical fluid equipment; extract with supercritical CO_2_ containing 10% ethanol as a co-solvent; perform LC-QTOF-MS/MS analysis on the extracted solid residue cake	Defatting rate is 86.75%, higher than that of the Soxhlet method (9.18%); the residual cake protein content increased to 74.4 g/100 g (68.4 g/100 g for the Soxhlet method)
Microwave-assisted extraction (Indirect)	Protein fragments and peptides generated by subsequent enzymatic digestion	*Chlorella vulgaris*	Prepare sample suspension and heat via microwave treatment until the suspension boils, followed by enzymatic hydrolysis	Increase the release of 32–40 kDa proteins; enhance peptide yield; promote subsequent enzymatic hydrolysis and peptide generation as a preprocessing method
Microbial fermentation	SGAVGEGAGGAGHPFFAPPQGW, PAEHPIL and seven other bioactive peptides	*Pyropia* spp.	Lactic acid bacteria consortium fermented in solid state at 37 °C, ultrasonically lysed and analyzed by LC-MS/MS; bioinformatic screening	Produce 58 diverse bioactive peptides (environmentally sustainable); improve protein solubility; optimize amino acid composition
Chemical synthesis (SPPS)	Aplidine analogue 4a, Tamandarin A analogue 5a	Synthetic	Synthesis of side-chain pseudo-dipeptides, cyclopeptide coupling, HPLC purification	Flexible structural modification; conformational restriction studies; preparation of high-purity products; scalability to support large-scale production
Structural optimization	WGWGW	*Ostrea rivularis Gould*	Molecular docking-guided amino acid substitution	Enhance xanthine oxidase inhibition over parent peptide

## Data Availability

This study did not create or analyze new data. This article does not apply to data sharing.

## Supplementary Materials

The following supporting information can be downloaded at: https://www.mdpi.com/article/10.3390/md24050170/s1, Table S1: Colorectal cancer cell lines and their representative molecular features; Table S2: In vivo anti-CRC efficacy of representative marine peptides.
