# Supplementary material for: Marine Bioactive Peptides for Colorectal Cancer Therapy: Mechanisms, Therapeutic Potential, and Translational Challenges
Source: Mar Drugs. 2026 May 9;24(5):170. doi: 10.3390/md24050170 (PMC13208497; doi:10.3390/md24050170)
Supplement: Supplementary file 1 [file marinedrugs-24-00170-s001.zip › marinedrugs-4180322-supplementary.pdf]

Supplement Table S1. Colorectal cancer cell lines and their representative molecular features.

CRC cell line	Molecular features
HCT116	KRAS G13D; PIK3CA H1047R; CTNNB1 mutation; MSI-H/dMMR phenotype; BRAF wild-type
HT-29	BRAF V600E; APC mutation; TP53 mutation; PIK3CA mutation; generally MSS/pMMR
DLD-1	KRAS G13D; PIK3CA E545K; APC mutation; TP53 mutation; MSI phenotype
SW480	KRAS G12V; APC mutation; TP53 mutation; SMAD4 alteration; MSS phenotype
SW620	KRAS G12V; APC mutation; TP53 mutation; SMAD4 alteration; metastatic lymph node-derived CRC cell line; MSS phenotype
LoVo	KRAS G13D; MSI-H/dMMR phenotype; BRAF wild-type
Caco-2	APC mutation; TP53 alteration; generally KRAS/BRAF wild-type
RKO	BRAF V600E; MSI-H/dMMR phenotype; CIMP-high; KRAS wild-type; PIK3CA mutation
HCT-8	KRAS-mutant background; MSI phenotype
26-L5	Murine colon carcinoma subline; KRAS-mutant background
SW948	KRAS-mutant background; BRAF wild-type

MSI-H, microsatellite instability-high; dMMR, deficient mismatch re pair; MSS, microsatellite stable; pMMR, proficient mismatch repair; CIMP, CpG island methylator phenotype. Molecular features were curated using Cellosauru (https://www.cellosaurus.org/).

Supplement Table S2. In vivo anti-CRC efficacy of representative marine peptides.

Peptide	Model	CRC cell line/model	Dose	Route	Frequency	Duration	Main efficacy	Toxicity/safety

rAj-HRP	Nude mouse xenograft	HCT 116	200 μg/kg	Intraperitoneal injection	Daily	14 days	58.41% tumour inhibition	Minimal toxicity: mouse weight changes similar to model group; significant weight decrease observed in 5-Fu (10 mg/kg) control group
P6	Nude mouse xenograft	HT-29	30 mg/kg	Subcutaneous injection	To be verified	14 days	72.66% tumour inhibition	No obvious toxicity reported; organ indexes of heart/spleen/liver/kidney showed no significant difference from control group
Ohmyungsamycin A	HCT116 subcutaneous xenograft nude mouse model	HCT 116	5 mg/kg; 10 mg/kg	Intraperitoneal injection	Three times per week	25 days	Tumour growth inhibition 5 mg/kg group: 70.3%;10 mg/kg group: 47.9%	No significant body weight loss or overt toxicity observed
Trichodermamide B	Xenograft model	HCT 116	20 mg/kg	Intraperitoneal injection	every day	14 days	65% tumour inhibition	No notable changes in body weight; no observable spleen and kidney injuries
Asperphenin A	Xenograft model	RKO or CRC xenograft model	4/8 mg/kg	Intraperitoneal injection	Three times per week	21 days	38.9%/68.7% tumour growth inhibition	No overt toxicity or body weight change; reduced body weight in irinotecan-treated groups

Desmethoxymajusculamide C, DMMC	Xenograft model	HCT 116	0.62 mg/kg/day	Intravenous injection	Daily	5 days	~60% tumour proliferation inhibition	MTD around 2.5 mg/kg (SCID mice); No severe toxicity reported at 0.62 mg/kg/day
Largazole	HCT116 xenograft mouse model	HCT 116, HT29, HCT 15	5 mg/kg	Intraperitoneal injection	Daily	Until control group reached endpoint (tumour length ≥15mm or volume ≥1000mm^3)	Inhibition of tumour growth and induction of apoptosis	Well tolerated up to 50 mg/kg i.p. with no acute toxicity
Cyclo(L-Pro-L-Phe) DKP-3	Zebrafish xenograft	HT-29	200 μM	Microinjection into embryo yolk sac	Single injection with 72 hours observation	72 hours	81% tumour inhibition	No embryotoxicity or normal cell cytotoxicity
Cyclic dipeptides from *Exiguobacterium acetylicum* S01 or related marine bacteria	Zebrafish xenograft	HT-29	To be verified	Direct exposure via water containing DKPs	Continuous exposure (0-72 hours)	At least 72 hours	>60% tumour-volume reduction	No obvious embryotoxicity (no significant decrease in survival rate or developmental toxicity/teratogenic effects)

i.p.,Intraperitoneal injection;MTD,Maximum Tolerated Dose;SCID,Severe Combined Immunodeficiency.
